# Laser-accelerated electron beams at 1 GeV using optically-induced shock injection

**DOI:** 10.1038/s41598-023-38805-3

**Published:** 2023-07-19

**Authors:** K. v. Grafenstein, F. M. Foerster, F. Haberstroh, D. Campbell, F. Irshad, F. C. Salgado, G. Schilling, E. Travac, N. Weiße, M. Zepf, A. Döpp, S. Karsch

**Affiliations:** 1 Ludwig-Maximilians-Universität München, Centre for Advanced Laser Applications, 85748 Garching, Germany; 2 Department of Physics, University of Strathclyde, Glasgow, G4 0NG UK; 3 Friedrich-Schiller-Universität Jena, Institut für Optik und Quantenelektronik, 07743 Jena, Germany; 4 Helmholtz-Institut Jena, 07743 Jena, Germany; 5 Max Planck Institut für Quantenoptik, 85748 Garching, Germany

**Keywords:** Laser-produced plasmas, Plasma-based accelerators

## Abstract

In recent years, significant progress has been made in laser wakefield acceleration (LWFA), both regarding the increase in electron energy, charge and stability as well as the reduction of bandwidth of electron bunches. Simultaneous optimization of these parameters is, however, still the subject of an ongoing effort in the community to reach sufficient beam quality for next generation’s compact accelerators. In this report, we show the design of slit-shaped gas nozzles providing centimeter-long supersonic gas jets that can be used as targets for the acceleration of electrons to the GeV regime. In LWFA experiments at the Centre for Advanced Laser Applications, we show that electron bunches are accelerated to ${1}\text {GeV}$ using these nozzles. The electron bunches were injected into the laser wakefield via a laser-machined density down-ramp using hydrodynamic optical-field-ionization and subsequent plasma expansion on a ns-timescale. This injection method provides highly controllable quasi-monoenergetic electron beams with high charge around ${100}\text {pC}$, low divergence of ${0.5}\text {mrad}$, and a relatively small energy spread of around ${10}\%$ at ${1}\text {GeV}$. In contrast to capillaries and gas cells, the scheme allows full plasma access for injection, probing or guiding in order to further improve the energy and quality of LWFA beams.

## Introduction

Laser wakefield acceleration (LWFA) is a promising candidate for future compact accelerator designs^1^. The high accelerating fields of laser-driven plasma wakefields of around ${100}\text {GV}/\text {m}$ at plasma densities of around $10^{18}\text {cm}^{-3}$ surpass the material breakdown limit of conventional radio-frequency accelerators by three orders of magnitude^2^. This facilitates the reduction of the accelerator size. For about a decade, research on LWFA has yielded ultrashort electron beams with energies of a few GeV^3–6^. Such beams may one day be suitable for generating high-energy x-rays and $\gamma$-rays, for example, in the next generation^7,8^ of free electron lasers (FELs)^9^. To be usable as reliable electron sources in the future, LWFA accelerators need to deliver stable, high quality beams, tunable up to the GeV level and beyond. To reach electron energies of several GeV with LWFA, acceleration needs to be sustained over centimeter-scale distances. High electron energies of up to  ${8}\text {GeV}$^10^ have been reached by using capillary discharge waveguides, which guide the laser in a preformed plasma channel generated by a discharge current^11,12^. Other external guiding structures, such as hydrodynamic optically-field-ionized (HOFI) channels^13,14^, have resulted in up to  ${5}\text {GeV}$^15^. However, also by relying on self-guiding of the laser pulse, energies in the GeV regime have been reached using gas cells^3,4,6^ and gas jets^5^ with no external guiding structure.

To achieve stable accelerating conditions, both the driving laser pulse and the gas target need to exhibit as few fluctuations as possible. Properly designed gas cells can provide a very homogeneous and reproducible density profile^16^, which is important for the stable acceleration of electron beams over a few centimeters. However, they suffer from laser-induced erosion of the entrance pinholes after each laser shot, which results in increasing density gradients over time^17^. If the gradient length surpasses the Rayleigh length, out-of-focus beam profile fluctuations start jeopardizing the stable propagation of the laser pulse.

In GeV experiments using gas cells, injection mechanisms such as self-injection^18^ and ionization injection^3^ have been applied to inject electrons into the laser wakefield. Both techniques can reliably inject electrons at the back of the wakefield bubble. This enables acceleration to high energies due to the full exploitation of the dephasing length^19^. However, both injection mechanisms have varying levels of control and often result in continuous injection and broadband energy spectra.

To inject electrons in a controlled and tunable way, density down-ramp injection can be used^20^. This method is often applied with supersonic gas jets, where a shock-front is created by introducing an obstacle in the supersonic gas flow^21,22^. The shock-front induces a rapid change in the plasma wavelength $\lambda _p$, which is inversely proportional to the plasma density $n_e$ according to $\lambda _p = 2\pi c\sqrt{\frac{\epsilon _0 m_e}{e^2n_e}}$. In the density drop of the shock, the plasma wavelength increases suddenly, and electrons, located at the back of the wakefield bubble before the density transition, can now be trapped inside the accelerating fields. This process is locally confined to the sharp gradient, which is a prerequisite for low-energy-spread electron bunches.

In comparison to gas cells, supersonic gas jets are less susceptible to laser-induced erosion. Furthermore, a direct measurement of the plasma density by interferometry is easier with gas jets, where a perpendicular probe beam can propagate freely through the gas profile without the need for windows. However, for the acceleration of electron beams to the GeV regime and beyond, the acceleration length of LWFA targets has to be on the scale of a few centimeters. Gas cells can easily be scaled to provide a longer interaction length. For conventionally round nozzles creating supersonic gas jets a simple scale-up for a given target gas density will, however, cause the gas load in the chamber, which scales as the square of the throat diameter, to become overly large for many vacuum systems. Keeping the same throat diameter while increasing the nozzle exit size will quickly lead to high Mach numbers and consequently an excessive density ratio before and after the shock. The most straightforward way to reach a long interaction distance while circumventing these issues is to reduce the dimensionality of the flow, i.e. going to slit-shaped nozzles.

For this work, computational fluid dynamics (CFD) simulations have been conducted to design slit-shaped nozzles a few cm long and a few mm wide, thus providing a long acceleration length over the longitudinal direction. A conical profile in longitudinal direction enables a supersonic flow and allows density down-ramp injection by introducing an obstacle into the flow. This method was recently used with a similar slit nozzle to generate quasi-monoenergetic electron beams around ${1}\text {GeV}$ in a preformed plasma waveguide^23^. In our work, a different method to generate a density down-ramp was applied that can also be used for controlled injection in a gas cell. We generate the density down-ramp by an evolution of a HOFI^13,24,25^ plasma gradient. To form the HOFI plasma gradient, a second laser beam is focused into the gas target between 0.2 and ${2}\text {ns}$ before the driver (for details, see the "Methods" section). A similar method was previously used for density down-ramp injection resulting in electron bunch energies up to ${400}\text {MeV}$^26–28^. In contrast to previous works, we used a spherical mirror under an angle to generate a highly astigmatic focus. Thus, with an easy alignment procedure, a line focus is generated, which is oriented perpendicular to the driver beam and mitigates possible pointing instabilities between the driver and the injector beam. Our group recently used the same technique to inject electrons into a beam-driven wakefield in a hybrid LWFA-PWFA scheme, demonstrating superior performance over purely hydrodynamic shocks^29^.

The combination of the newly designed ${2}\text {cm}$ long slit nozzle and the laser-machined down-ramp injection resulted in quasi-monoenergetic bunches around ${1}\text {GeV}$ with high charge, low divergence and small energy spread. We show that these ${1}\text {GeV}$ electron beams can be tuned by moving the position of the density down-ramp and adjusting the properties of the shock by changing the delay between the injector beam and the driver laser.

## Slit nozzle design

**Figure 1:**
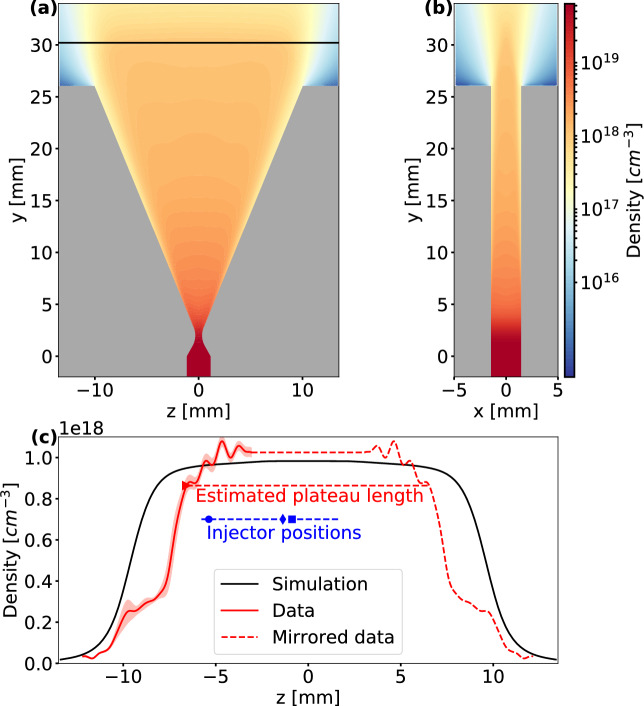
Electron density distributions of slit nozzle. (**a,b**) Simulated electron density distributions at cuts through the center of the nozzle along the longitudinal and transverse direction, respectively. The black line indicates the propagation of the laser with ${4.2}\text {mm}$ distance to the nozzle exit. (**c**) Density line-outs. The black solid line shows the line-out of the simulated electron density distribution along the laser propagation axis. The red solid line is the mean of five interferometry measurements of the plasma electron density in experimental conditions. The standard deviation of the measurements is indicated in transparent red. The red triangle marks the defined start of the density plateau. The red dashed line indicates mirrored and extrapolated data to form a complete profile, which is assumed to be symmetric to the center of the nozzle (details in "Methods"). From this, the estimated plateau length can be deduced. The location of the injector beam for different data sets in the results section is indicated in blue: Circle for data in Fig. 3a, diamond shape for data in Fig. 3b and square for data in Fig. 4b. The dashed blue line indicates the range for the scan in Fig. 4a.

CFD simulations were conducted to design slit nozzles intended as gas targets for the acceleration of electrons to GeV energies. The LWFA drive beam will be oriented along the long axis of the slit, which therefore defines the interaction distance. The slit width is large enough to avoid a strong density gradient in the flow direction, which otherwise would refract the drive laser beam into a curved beam path. In this work, a slit nozzle with a transverse extent of ${3}\text {mm}$ and a convergent-divergent shape in longitudinal direction was designed. Along the longitudinal direction, the nozzle has an extent of ${750}\upmu \text {m}$ in the throat, diverging to an exit length of ${20}\text {mm}$. The Mach number at the exit of the nozzle, defining the speed of the supersonic gas flow, is $M=5.15$ for hydrogen^30^.

Results of the 3D-CFD simulation for this nozzle can be seen in Fig. 1. Here, the electron density distribution is shown for a longitudinal and a transverse cut through the center of the nozzle. The simulation was carried out with an inlet pressure of ${2.6}\text {bar}$, corresponding to the inlet pressure used in the experiments.

In Fig. 1c a line-out of the simulated electron density is shown for a height of ${4.2}\text {mm}$ above the nozzle, which corresponds to the laser propagation height in the experiments. The CFD simulation shows a plateau density of ${0.97} \times 10^{18}\text {cm}^{-3}$ at this height.

The nozzle design was 3D-printed from the photopolymer material VeroWhitePlus$^{TM}$ (RGD835) and used as a gas target in LWFA experiments. The actual plasma electron density of the plateau was measured directly in the LWFA setup using interferometry. Beforehand, the interferometric measurements had been compared to plasma wavelength measurements^31^ and found to be consistent with them. In Fig. 1c the measured density is plotted alongside the simulated line-out. The measurements were conducted at an inlet pressure of ${2.60} \pm {0.02}\text {bar}$ and a distance of ${4.2} \pm {0.1}\text {mm}$ between laser axis and nozzle. The field of view of the interferometry imaging setup is smaller than the length of the slit nozzle to accomplish higher resolution. Therefore, only the density gradient at the entrance of the gas jet and the beginning of the plateau was measured. The beginning of the plateau is defined to start at ${80}\%$ of the measured peak density and is indicated in Fig. 1c with a red triangle. The density in the plateau was approximated by measuring the mean density of the first ${4.8}\text {mm}$ of the plateau. The measured density of ${1.0} \pm {0.2} \times 10^{18}\text {cm}^{-3}$ is close to the simulated plateau density at the same inlet pressure and height above the nozzle. Assuming a symmetric profile above the nozzle, the measured data was mirrored and extrapolated. From this, the plateau length could be estimated to be ${13.1}\text {mm}$ (for details see "Methods" section).

As can be seen in the graph, the measured density gradient exhibits a step around ${-9}\text {mm}$ that is not present in the simulated density line-out. Since the nozzle was a prototype and as such 3D-printed, it is possible that the surface roughness of the inside face in the diverging part of the nozzle was not as good as what could be achieved with nozzles fabricated from stainless steel. Therefore, we assume that the density step in the measured flow was caused by a turbulence layer and flow separation^32^. Due to its rather low density and limited extent, we conclude that this step has only a minor influence on the laser propagation properties. The surface roughness of the printed nozzle could also be an explanation for the oscillations that can be seen in the plateau. On the other hand, these oscillations could be noise from the interferometric measurement (see "Methods" for details).

**Figure 2:**
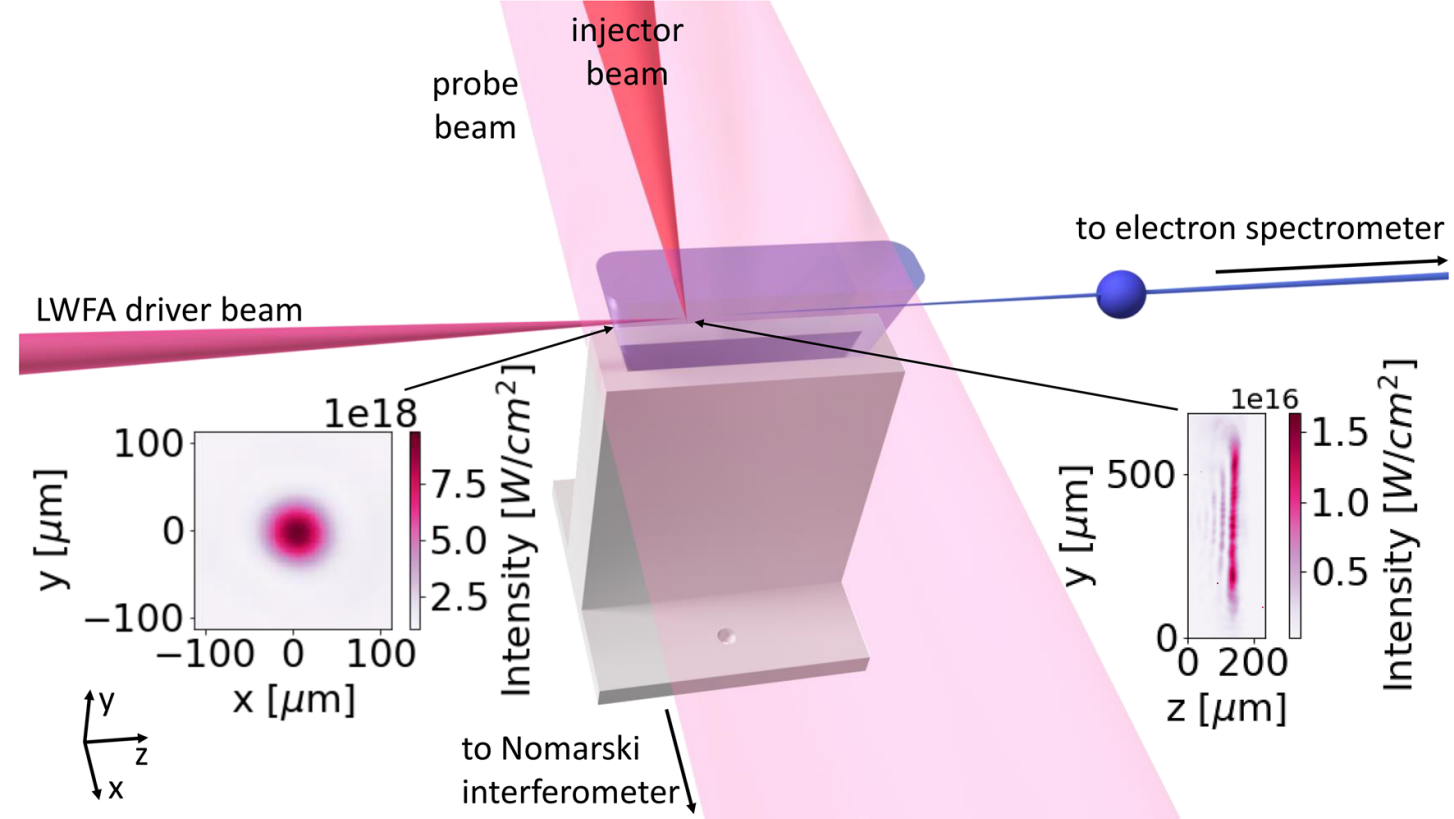
Schematic of the experimental setup showing the slit nozzle as gas target. The LWFA laser drives a wakefield in the gas jet. The left and right insets depict the measured vacuum focus of the driver beam and the astigmatic line focus of the injector beam, respectively. The latter is oriented perpendicularly to the drive beam axis and arrives between 0.2 and ${2}\text {ns}$ early, allowing the plasma to evolve before the drive beam arrives. At the density transition generated by the injector beam, an electron bunch is injected into the wake. A large diameter probe beam propagating perpendicular to the driver beam is used to measure the plasma density using a Nomarski interferometer. The injected electron bunch is accelerated in the wakefield and travels further downstream to an electron spectrometer.

## Experimental results

**Figure 3:**
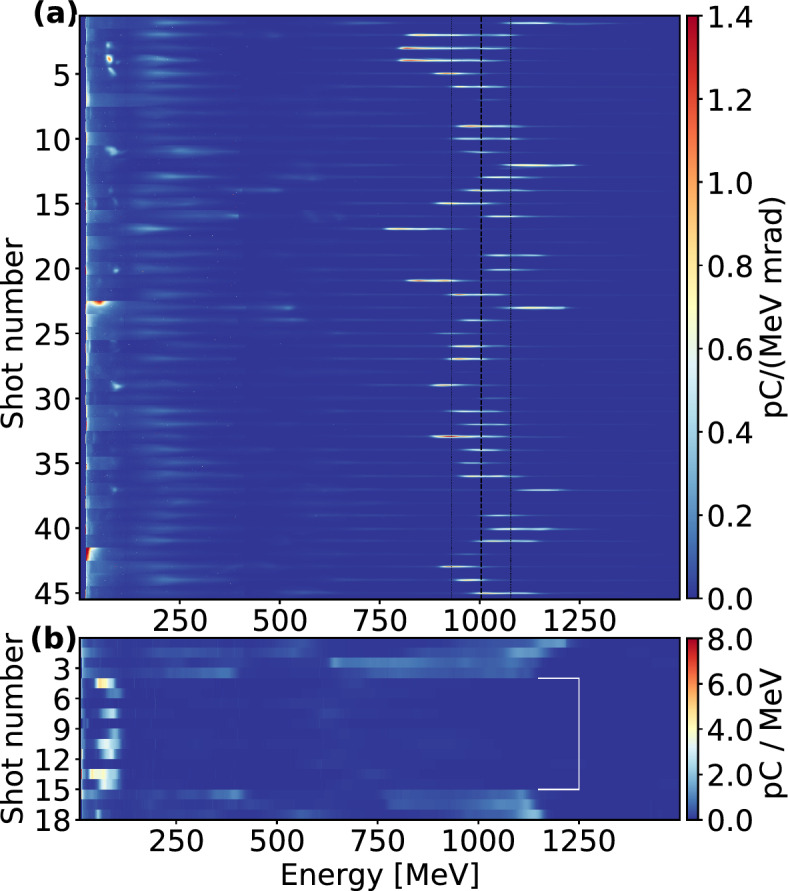
Data from LWFA experiments. (**a**) A set of 45 consecutive shots shows electron beams injected at the HOFI-generated density down-ramp. The mean center of mass energy of the set and the standard deviation are indicated with black dashed lines. (**b**) A set of 18 shots is shown with the injector beam being switched on and off. The white bracket indicates the shots without injector beam. In (**a**) the results are plotted divergence-resolved ranging from $\pm {5.4}\text {mrad}$, while in (**b**) the data for each shot is integrated over the width of the detector focal plane.

**Figure 4:**
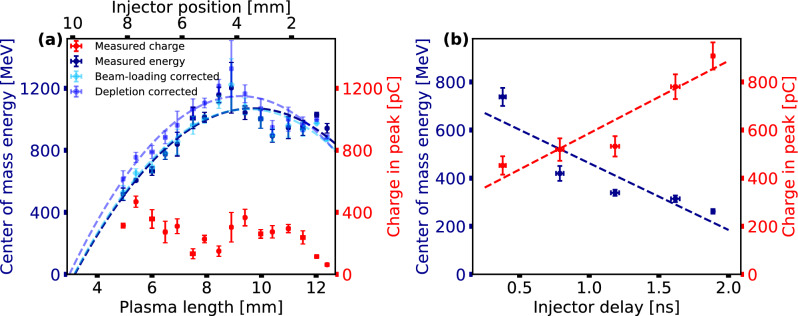
Two scans of experimental parameters. (**a**) The gas nozzle was moved in main laser direction with respect to both the injector plane and the drive beam focus. This changes the available plasma length in the plateau, against which the measured center of mass energy and the charge within a range of ${20}{\%}$ of the peak spectral charge of the injected bunches are plotted in dark blue and red, respectively. Here, the injector pulse arrived ${270}\text {ps}$ before the main beam and the plateau density was ${1.0} \times 10^{18}\text {cm}^{-3}$. In lighter blue tones the energy values are plotted corrected for beam-loading and depletion. (**b**) Dependence of energy and charge of the injected bunches on injector pulse timing. The injector position in this scan was ${5.7}\text {mm}$ from the plateau beginning, corresponding to a remaining plasma length of ${7.4}\text {mm}$, and the density in the plateau was ${1.1} \times 10^{18}\text {cm}^{-3}$. Measurements for close injector positions or delay values, respectively, were binned, and their mean values were plotted. The error bars show the standard error in injector position or injector delay, respectively, and energy and charge.

A schematic drawing of the experimental setup is presented in Fig. 2. The slit nozzle described in the previous section was used to generate a gas jet as target for the LWFA experiments. The injector beam contains approximately ${90}\text {mJ}$ and propagates in x-direction, perpendicular to the drive beam axis in z-direction. It generates a plasma sheet in the x-y plane. In the time before the main pulse arrives, this hot plasma sheet expands to two nearly planar shock regions (see "Methods"). At the down-ramp of the second shock, electrons are injected into the laser wakefield.

In Fig. 3a results of accelerated electron bunches are shown. These electron beams were obtained at a plasma density of ${1.0} \pm {0.2} \times 10^{18}\text {cm}^{-3}$, an injector beam position of ${1.2} \pm {0.1}\text {mm}$ from the beginning of the plateau, and a delay between the injector beam and the main beam of ${270} \pm 10\text {ps}$. The set contains 45 consecutive shots of electron beams with a center of mass energy of ${1.003} \pm {0.074}\text {GeV}$ and an energy spread of ${94} \pm {27}\text {MeV}$. Within a range of ${20}{\%}$ of the peak spectral charge, the bunches contain a charge of ${92} \pm {56}\text {pC}$ and show a Gaussian-fitted RMS divergence of ${0.49} \pm {0.05}\text {mrad}$. As demonstrated in other scans (summarized in Fig. 4), energy and charge can be individually optimized to higher values. However, aiming for stable beams with a small bandwidth at the GeV level, we identified this regime as favorable at our fixed laser parameters due to the combination of high energy of ${1}\text {GeV}$ with good energy stability of ${7}{\%}$ and a small bandwidth of under ${10}{\%}$.

These results demonstrate that acceleration can be sustained long enough to reach ${1}\text {GeV}$ without the need for an external guiding structure using a long vacuum Rayleigh length of ${9.3}\text {mm}$ and self-guiding. The high laser energy in the experiment of ${6}\text {J}$ on target yielded high charge beams with nearly ${100}\text {pC}$ in the peaks at ${1}\text {GeV}$. While the total charge on the whole spectrometer, measuring between ${12}\text {MeV}$ and ${1.5}\text {GeV}$, was ${306} \pm {83}\text {pC}$, yielding a total energy conversion efficiency of ${2.3}{\%}$, most laser energy was converted into the high energy electron beam, as an energy conversion efficiency of ${2.0}{\%}$ was reached over ${350}\text {MeV}$ (total charge over ${350}\text {MeV}$: ${135} \pm {58}\text {pC}$). The measured energy conversion efficiency is comparable to recent works^23^, where lower laser energy but a smaller spot size was used, and an external guiding structure was applied to overcome the limit of diffraction. In this way, electron beams of similar energy, bandwidth and divergence were generated, but containing lower charge compared to our results.

In Fig. 3b we identify the optically generated shock-front as the origin of the injection. After the first 4 shots, the injector beam is switched off, after which no electron beams around ${1}\text {GeV}$ are injected. For the 11 shots without the injector beam, a total charge on the spectrometer of ${144} \pm {81}\text {pC}$ was measured, corresponding to a total energy conversion efficiency of ${0.6}{\%}$. After shot 15 of the set, the injector beam is switched back on, and injection around ${1}\text {GeV}$ resumes. This result indicates the influence of the plasma gradient for controlled electron injection. The data in Fig. 3b was taken at almost the same parameters as in Fig. 3a, only the shock position was moved further downstream to ${5.2}\text {mm}$ from the beginning of the plateau.

As shown in Fig. 3b, injection is controlled by the injector beam and results in quasi-monoenergetic electron bunches due to the local injection point. Using this, the injection point can be arbitrarily moved along the gas plateau length, and thus the remaining plasma length in the plateau can be varied. This has direct influence on the final energy of the injected bunches. However, in a density distribution that is not completely uniform, also the injected charge could change, which can influence the final energy via beam-loading^33,34^. This is illustrated in Fig. 4a, where the influence on energy and charge of a scan of the injector beam position (remaining plasma length) is shown. Assuming a plateau length of $L_{pl} = {13.1}\text {mm}$ from interferometry measurements (see Fig. 1c and "Methods"), the remaining plasma length $L_{rp}$ inside the plateau can be calculated by $L_{rp} = L_{pl} - L_{I}$ with $L_I$ being the length between the beginning of the plateau and the position of the injector beam, which was also deduced from interferometry images.

The directly measured electron energy values for the injector position scan (see Fig. 4a) are compatible with the parabolic shape expected for acceleration in the bubble regime^35^. The fit yields a maximum acceleration gradient (slope of the parabola at zero-crossing) of ${329}{\text {GV}/\text {m}}$, a dephasing length (length from zero-crossing to maximum) of ${6.5}\text {mm}$ and a maximum electron energy of approximately ${1.1}\text {GeV}$. However, while in qualitative agreement, we find that both energy gain and estimated dephasing length differ considerably from theoretical estimates based on electron acceleration in the blow-out regime^19^. For our parameters, the theoretical maximum energy gain at a matched normalized vector potential of $a_0 = 3.7$ is estimated at ${2.1}\text {GeV}$ and the dephasing length is ${23.8}\text {mm}$, which both exceed the observed values considerably. However, the scaling law from Lu et al.^19^ can only be viewed as an estimate of possible energy gain at our laser power. A direct comparison is difficult due to unmatched guiding conditions and a laser pulse length that is shorter than the spatial width of the laser, yielding a longer dephasing length than the depletion length of the laser. This contradicts the condition for Lu et al.’s scaling law. Therefore, a discrepancy in energy gain between Lu et al.’s predictions and our results is to be expected. Nonetheless, in the following, we will discuss a few effects that also play a role in limiting the energy gain, such as beam-loading, depletion and the injection phase, and will attempt to explain the much smaller measured dephasing length.

To estimate the influence of some of these effects on the energy gain, we correct the measured energy for beam-loading and laser depletion effects. First, we estimate the beam-loading contribution from analyzing Fig. 3a, yielding an electron energy - charge dependence $E(Q) = (1037 \pm 22)MeV - (0.37 \pm 0.21)MeV \times Q[pC]$. This correction is plotted along with the raw data in Fig. 4a, but only gives rise to a minor modification of the acceleration gradient (${334}{\text {GV}/\text {m}}$). Second, it is necessary to account for the laser depletion during its propagation through the jet before the injection point. This is due to the fact that for optical shock generation, in contrast to standard blade-induced shock injection, the gas flow is not disrupted by the blade before the injection point. The reduced laser power $P'$ for each injection point is calculated according to $P' = P \left( 1- \frac{L_{I}}{L_{depl}}\right)$ with the theoretical depletion length for our parameters $L_{depl} = {15.7}\text {mm}$. Using $P'$, we calculated a relative change in the theoretically expected electron energy and applied it to our measured energy values. The expected correction in electron energy is also plotted in Fig. 4a. From the corresponding fit, corrected values for the acceleration gradient (${368}{\text {GV}/\text {m}}$), dephasing length (${5.2}\text {mm}$) and maximum energy gain (${1.15}\text {GeV}$) are obtained. This analysis indicates that both beam-loading and depletion yield noticeable, yet minor reductions in energy gain. As discussed above, a direct comparison to the energy gain calculated by Lu et al. is difficult due to the above-mentioned conditions in the scaling law (matched spot size and matched pulse length). However, we also measure a much smaller dephasing length than theoretically calculated. This points to another limiting factor in energy gain, namely the injection phase. Injection at a density down-ramp causes the bulk of the injected electron population in the first wakefield bubble to be trapped at a position further inside the bubble. Using the same framework as used for rephasing in Ref.^35^, we estimate this ”phase” position as $\phi = 2 \pi (1- R_{1}/R_{2})$, where $R_{1}$ and $R_{2}$ denote the bubble radius before and after the shock, respectively. The phase in the bubble is defined to be 0 at the back of the bubble and $2\pi$ at the laser position. From this estimate, we can see that the injection phase can be relatively close to the dephasing point at $\pi$ for a high density step. Therefore, for high shock density ratios, the injected electron beams miss the highest accelerating fields at the back of the bubble, and the dephasing length is reduced by this injection phase advance, leading to significantly lower energy gain.

In Fig. 4b a further advantage of generating the shock with a laser beam is illustrated. The properties of the shock can be tuned by changing the delay between the injector beam and the LWFA driver laser. The earlier the injector beam arrives, the longer the plasma sheet expands with the speed of sound^29^ influencing the height of the shock and enabling more electrons to be injected. Therefore, a clear dependence of the injected charge on the injector delay is seen in Fig. 4b. Due to the astigmatic line focus used in this work, we could mitigate pointing instabilities between the injector beam and the driver beam and could increase the probability of injecting with small delays to over ${90}{\%}$ (see Fig. 3a). Previous works^26^ saw with increasing delay mainly a higher probability of injecting a bunch into the wakefield. The constantly high injection probability in our experiment allowed for better statistics over the entire scanning range.

With increasing delay, a decrease in energy of the injected bunches is also observed, which can be partly attributed to beam-loading due to the increasing charge. Due to higher inlet pressure, the density was with $1.1 \times 10^{18}\text {cm}^{-3}$ slightly higher than for the data in Fig. 3 and Fig. 4a, explaining the overall higher injected charge, and thus lower energy due to beam-loading. Also, the advancing injection phase with an increasing shock density ratio at longer delays might contribute to the observed decrease in energy. From the data obtained so far, it is difficult to estimate which of these two effects is dominant. Further measurements to answer this will be the object of further investigations.

## Outlook

We have shown that our design of a supersonic slit nozzle can be used as a LWFA target for the acceleration of electrons to ${1}\text {GeV}$. We used a laser beam for generating a HOFI plasma gradient to enable and control density down-ramp injection. This resulted in stable quasi-monoenergetic electron beams with low divergence at ${1}\text {GeV}$. With a long Rayleigh length, these results can be obtained without the need for a guiding structure. The high laser energy that was used results in high charge bunches around ${100}\text {pC}$. In future experiments, the laser energy will be further increased to investigate if high quality bunches can be generated at higher energies, still without a guiding structure. External guiding structures such as HOFI channels as used in previous studies^13,14,23^ will be implemented eventually. However, this requires active pointing stabilization, which is currently being developed for the ATLAS-3000 laser system. With a guiding structure, higher electron energies could already be reached with current laser parameters, as was recently shown by Miao et al.^15^. In the future, the controlled injection scheme using HOFI plasma gradients might be used to generate electron bunches with energies of a few GeV. Using active laser stabilization^36^, the electron beam stability could be further improved. These beams could then be used for radiation reaction experiments^37^ or the generation of $\gamma$-ray bunches for a Breit-Wheeler pair production experiment^38–40^, where high charge bunches at a few GeV with low divergence are needed.

## Methods

### CFD simulations

For the CFD simulations, the geometry of the gas flow volume in 3D inside the nozzle was drawn and meshed into small simulation volumes of sizes between $6.3 \times 10^{-5}\text {mm}^3$ and $7.6 \times 10^{-3}\text {mm}^3$. Here, drawing a quarter of the geometry is sufficient due to the symmetry of the nozzle. CFD simulations were conducted with the software ANSYS Fluent. The Fluent software works on the basis of solving conservation equations for mass and momentum. In the case of compressible flows, as applicable to supersonic gas flows, energy conservation is also taken into account^41^. The simulations were conducted using the density-based solver, which uses the continuity equation to retrieve the density field and resolves the pressure field using the equation of state. For turbulence, the SST k-$\omega$ viscous model was used. The gas flow was simulated with hydrogen as gas species as was used in the LWFA experiments of this work. The ideal gas law was assumed for the relation between gas density, temperature and pressure. For the simulated line-out shown in Fig. 1c, points were interpolated between simulation points.

### Density measurements

A probe beam was picked from the main driver beam before focusing and sent perpendicularly through the gas jet. At the target, the probe beam has a size of ${1}{''}$. The gas jet was imaged with a 1.1x magnification. The probe beam with a center wavelength of ${800}\text {nm}$ was then sent through a Nomarski-prism to generate an interferogram on a 12-bit CMOS camera. The spatial resolution of the system was estimated to be ${25}{\mu } \text {m}$. The interferogram was analyzed using Fourier filtering to filter out noise. Here, a trade-off between filtering out noise and losing information has to be found. With smaller filtering windows, the oscillations seen in the measured phase profile can be clearly reduced. However, this also caused the measured phase difference and consequently, the measured density of the plateau to be clearly reduced, indicating the loss of information. Therefore, the measurements seen in Fig. 1c use the smallest possible filtering window without losing information but with a remaining oscillation on the phase profile that could originate from noise. From the measured phase difference after Fourier filtering the density could be calculated via Abel inversion exploiting the symmetry of the plasma channel. The error of the interferometric measurement of the density plateau of ${20}{\%}$ was calculated from the standard deviation of five measurements, the standard deviation of the fluctuations in the plateau, and the fit errors from a linear fit to measurements at different inlet pressures. Since the field of view of the imaging system was approximately ${10}\text {mm}$ in length, the longer jet could not be fully imaged. Assuming the density profile to be symmetric above the nozzle, the plateau length could be approximated by measuring the distance from the start of the plateau to the nozzle edge seen in the interferometry images and subtracting twice this length from the known length of the nozzle.

### ATLAS laser system

The ATLAS-3000 laser at the Centre for Advanced Laser Applications (CALA) is a Ti:Sa laser system with a center wavelength of ${800}\text {nm}$ using chirped pulse amplification. After amplification, the pulses are recompressed to ${30}\text {fs}$ FWHM duration. For the experiments shown in this report, the ATLAS laser delivered laser pulses with ${6} \pm {1}\text {J}$ on target. Here, the large error margin has to be taken into account since the beamline mirrors and gratings undergo blackening and the specific transmission at the time of the experiments was not measured. Focused down with a F/56 spherical mirror a FWHM spot size of ${57} \pm {1}{\upmu } \text {m}$ was obtained, and a peak intensity of ${8.9} \pm 0.8 \times 10^{18}{\text {W}/\text {cm}^2}$ was reached corresponding to a normalized vector potential of $a_0 = 2.0$.

### Injector beam

To inject electrons into the wakefield, a laser beam is picked from the main beam before focusing and sent perpendicularly to the main beam through the gas target. The delay of the injector beam is adjustable to arrive between 0 to ${2}\text {ns}$ before the main beam and the beam contains ${90}\text {mJ}$ of energy. It is focused with a ${15}\text {cm}$ focal length spherical mirror under an angle of incidence of ${20}{\%}$, generating an astigmatic focus with a FWHM spot size of $({20}\times {450})\upmu \text {m}^2$ and a peak intensity of $1.6 \times 10^{16}{\text {W}/\text {cm}^2}$. As described in Foerster et al.^29^ the injector beam locally ionizes and heats the plasma. The heated electron population propagates away at the speed of sound, which is higher than the speed of sound of the neutral gas, leading to shock waves at the edge of the expanding region. At the density transitions of these shocks, electrons can be injected into the wakefield via density down-ramp injection.

### Electron beam diagnostics

The energy spectrum of the electron beam is diagnosed using a ${80}\text {cm}$ long dipole magnet with a magnetic field strength of ${0.85}\text {T}$. This magnet is situated ${2.9}\text {m}$ downstream of the LWFA target. The downward-deflected electrons impinge on a scintillating screen located below the magnet, and the calibrated fluorescence emission of the screen^42^ was imaged by a 12-bit CMOS camera to measure energy, charge and divergence of the electron beams. A second scintillating screen was placed before the entrance of the magnet and imaged onto a camera. From this, the pointing of the electron beams and thus the entrance location and angle of the beams into the magnet could be obtained and used for a more accurate analysis of the electron energy.

## Data Availability

The data shown in this report is available from the corresponding authors upon reasonable request.
